# Augmented exercise in hospital improves physical performance and reduces negative post hospitalization events: a randomized controlled trial

**DOI:** 10.1186/s12877-020-1436-0

**Published:** 2020-02-07

**Authors:** Ruth McCullagh, Eimear O’Connell, Sarah O’Meara, Darren Dahly, Eilis O’Reilly, Kieran O’Connor, N. Frances Horgan, Suzanne Timmons

**Affiliations:** 1 Centre for Gerontology & Rehabilitation, University College Cork, Cork, Ireland; 2 Physiotherapy Department, Mercy University Hospital, Cork, Ireland; 3 Clinical Research Facility, Mercy University Hospital, Cork, Ireland; 4 School of Public Health, University College Cork, Cork, Ireland; 5 Clinical Research Facility, University College Cork, Cork, Ireland; 6 Department of Geriatric Medicine, Mercy University Hospital, Cork, Ireland; 7 School of Physiotherapy, Royal College of Surgeons in Ireland, Dublin, Ireland

**Keywords:** Frail, Hospitalisation, Exercise, Physiotherapy, Length of stay

## Abstract

**Background:**

To measure the effects of an augmented prescribed exercise programme versus usual care, on physical performance, quality of life and healthcare utilisation for frail older medical patients in the acute setting.

**Methods:**

This was a parallel single-blinded randomised controlled trial. Within 2 days of admission, older medical inpatients with an anticipated length of stay ≥3 days, needing assistance/aid to walk, were blindly randomly allocated to the intervention or control group. Until discharge, both groups received twice daily, Monday-to-Friday half-hour assisted exercises, assisted by a staff physiotherapist. The intervention group completed tailored strengthening and balance exercises; the control group performed stretching and relaxation exercises. Length of stay was the primary outcome measure. Blindly assessed secondary measures included readmissions within 3 months, and physical performance (Short Physical Performance Battery) and quality of life (EuroQOL-5D-5 L) at discharge and at 3 months. Time-to-event analysis was used to measure differences in length of stay, and regression models were used to measure differences in physical performance, quality of life, adverse events (falls, deaths) and negative events (prolonged hospitalisation, institutionalisation).

**Results:**

Of the 199 patients allocated, 190 patients’ (aged 80 ± 7.5 years) data were analysed. Groups were comparable at baseline. In intention-to-treat analysis, length of stay did not differ between groups (HR 1.09 (95% CI, 0.77–1.56) *p* = 0.6). Physical performance was better in the intervention group at discharge (difference 0.88 (95% CI, 0.20–1.57) *p* = 0.01), but lost at follow-up (difference 0.45 (95% CI, − 0.43 – 1.33) *p* = 0.3). An improvement in quality of life was detected at follow-up in the intervention group (difference 0.28 (95% CI, 0.9–0.47) *p* = 0.004). Overall, fewer negative events occurred in the intervention group (OR 0.46 (95% CI 0.23–0.92) *p* = 0.03).

**Conclusion:**

Improvements in physical performance, quality of life and fewer negative events suggest that this intervention is of value to frail medical inpatients. Its effect on length of stay remains unclear.

**Trial registration:**

ClinicalTrials.gov Identifier: NCT02463864, registered prospectively 26.05.2015.

## Background

It is well established that older medical inpatients are minimally active in hospital. Patients walk an average of 600 steps daily [1, 2] which equates to 12 mins of walking [3]; 49% of older patients remain on bedrest or transfer from bed to chair only [4], and less than 19% of patients walk hospital corridors [5]. Our recently conducted observation study suggested that people who walked more had a shorter stay in hospital, where a 50% higher step-count was associated with a 6% shorter hospital stay, and those with poor physical performance on admission were the least active in hospital [1]. These frailer patients are potentially most at risk of functional decline following a hospital admission [6].

Interdisciplinary team care has been found to improve patients’ health outcomes and length of stay [7–11]. While effective, it requires a considerable investment and change in clinical practice. There is emerging evidence of acute sarcopenia secondary to hospitalisation, defined as a loss of muscle mass, loss of muscle strength and low physical performance, which has been linked to poorer quality of life (QoL), increased falls risks and increased mortality [12]. Therefore, a simple exercise programme could be easy to implement but effective in preventing acute sarcopenia. Trials which have included both robust and frail inpatients have shown limited effectiveness of exercise alone on length of stay [13, 14], with conflicting results on physical and functional performance [13, 15]. A meta-analysis of exercise interventions suggested that they were more effective for those patients needing more assistance to walk [16]; potentially as those patients are most at risk of acute sarcopenia [1]. Positive effects on functional and physical capacity were gained in an exercise intervention specifically for very elderly patients in hospital. The patients (*n* = 370) exercised in an equipped gym in the hospital [17]. However, the results of this study are not generalisable to hospitals with limited gym facilities. The question remains whether a simple exercise intervention with limited resources and access to exercise equipment can yield positive results.

Therefore, the primary aim of this trial was to measure the effectiveness of an augmented prescribed exercise programme (APEP) on frail older medical patients in the acute setting. The programme was delivered at the bedside, using body weight as the resistance, and included balance and walking within the programme. Its effectiveness on length of stay (as the primary outcome measure), physical performance and QoL at discharge and at three months’ post discharge, and readmission rates over the subsequent three months’ post discharge was measured.

## Methods

A detailed description of the APEP trial protocol has been presented previously [18]. The trial received ethical approval from the local clinical research ethics committee.

### Design

The study was a prospective, sham-intervention controlled, randomised trial, with blinded randomisation and outcome measurement. It was completed between March 2015 and January 2017.

### Patient selection and setting

Recruitment took place in one 350-bedded general teaching hospital. All wards admitted older medical inpatients, including one small geriatric ward. At the time of the trial, 17 medical consultants and four geriatricians were based in the hospital. Rehabilitation and general staffing levels were comparable across all wards. Irrespective of ward allocation, medical inpatients aged 65 and over, needing an aid and/or assistance to walk on admission, and admitted from and planned for discharge home (rather than for institutional care), with an anticipated hospital stay ≥3 days were recruited. The following patients were excluded: inpatients >48 h prior to screening; unable to follow simple commands in the English language; admitted with an acute psychiatric condition, or requiring end-of-life or critical care; ordered bedrest, or contraindications to walking (e.g. hip fracture or high ventricular rate atrial fibrillation); baseline Short Physical Performance Battery (SPPB) score 0/1; participated in the trial within the previous 12-months.

### Recruitment process

Recruitment to the trial was completely independent from routine physiotherapy referrals and services. Using the electronic hospital management system, the principal investigator (RMC) identified suitable patients. Patients were not recruited on Fridays as no exercises sessions were delivered over the weekend. The medical team confirmed their suitability prior to the patient being approached. Patients were verbally informed about the study, and questions were answered. Relatives were contacted by phone if requested. Cognitive impairment is highly prevalent in patients aged ≥70 years of age in acute care [19]. Therefore, we aimed to include patients who could exercise with one-to-one guidance. Those with significant cognitive impairment, who were unable to follow instructions and guidance were excluded. If cognition appeared or was reported as poor, their next-of-kin assented to their inclusion. All participants gave written informed consent. Patients were recruited as resources allowed; a maximum of five patients participated in the study simultaneously.

### Interventions

Both groups received usual care with additional exercises. The augmented prescribed exercise programme (APEP) was delivered to the intervention group, and a sham programme to the control group. The APEP programme aimed to improve strength, balance and walking, while the sham intervention was mainly breathing and stretching exercises. For a full description, please see Additional file 1: Appendix 1: Description of APEP and Sham Exercise Programmes. These additional exercises were performed twice-daily, Monday-Friday, and the sessions lasted up to 30 mins, (depending upon the patient’s exercise tolerance). At each session, contraindications were reviewed and verbal consent sought. Routine physiotherapy was not affected by the APEP trial and was delivered based on physiotherapy’s assessment of the patient and competing caseload, averaging three sessions per week. Patients in the acute stroke unit fitting the inclusion criteria were invited to participate. Patients with a major stroke met the exclusion criteria. They continued with their usual care, which was similar to other general medical patients until they were transferred to an offsite rehabilitation stroke unit.

### Descriptive measures

On admission, the patients’ demographics and medical history were noted. Their home situation, medication use, co-morbidity (Cumulative Illness Rating Scale-Geriatrics, CIRS-G [20]), cognition (Six Item Cognitive Impairment Test, 6CIT) [21] frailty, including grip strength (Survey of Health, Ageing and Retirement in Europe, SHARE-FI) [22], falls history over the previous 6 months, and falls efficacy (Falls Efficacy Scale International, FES-I) [23], were measured on initial assessment.

### Outcome measures

The assessment schedule is described in Table 1.

**Table 1:**
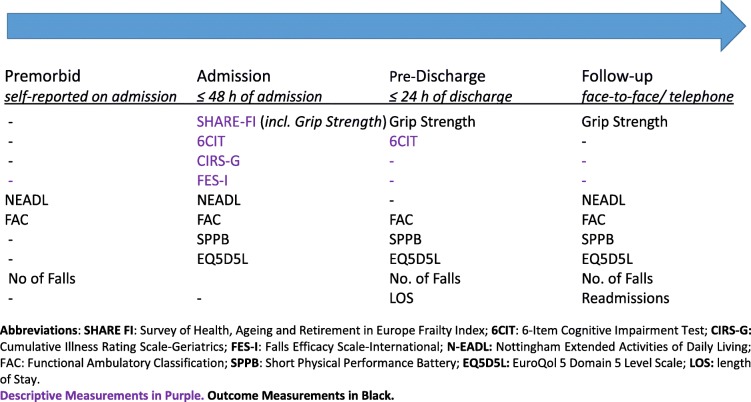
Descriptive and Outcome Measurements Assessment Schedule

The effects of the intervention on healthcare utilisation (length of stay and readmission rates), physical performance (SPPB) [24] and QoL (EuroQol 5 Domain 5 Level Scale, EQ 5D5L [25]) were measured. In addition, the effects on functional independence (Nottingham Extended Activities of Living, N-EADL [26]), functional ambulation (Functional Ambulatory Classification, FAC [27]), and falls rate, were also measured.

### Healthcare utilisation

The primary outcome measure was length of stay (bed nights) (LOS). The number of readmissions over the subsequent 3 months was also recorded; both data were readily available from the electronic hospital information system.

### Physical performance and daily activity

Functional Independence was measured using N-EADL [26], premorbidly, on admission, and at the three-month follow-up.

The SPPB [28] was used to measure physical performance on admission, at discharge and at follow-up. Walking speed was assessed over four metres and only in those who could walk without assistance (standby assistance without contact was provided as required).

The FAC [27] was used to measure their functional ambulation. Patients’ walking was observed on admission, at discharge and at follow-up. On admission, the patients were asked to self-report their premorbid ambulatory level. Self-report was also used at the follow-up when the patient couldn’t attend in person. While the FAC has not been validated as a self-reported tool, it did provide some information about their ambulatory level when observation was impossible.

Patients’ walking was continually measured using the Stepwatch Activity Monitor (SAM).

### Falls and QoL

Number of falls over the previous 6 months was self-reported on admission. In-hospital falls were recorded from hospital notes, while post discharge falls were self-reported at follow-up.

QoL was measured using the EQ 5D5L [25] on admission, discharge and at follow-up. The next-of-kin was asked to complete this if the patient was unable. The reliability of proxy reports has been debated with evidence suggesting that proxy reports are poorer than self-reports [29]. However, other studies have found little or no difference between self and proxy reports in older adults [30], patients with traumatic brain injury and Parkinson’s Disease [31], therefore, the decision to include proxy reports was made.

Changes in living arrangements (change in accommodation, support or home adaptations) were recorded at discharge and follow-up.

### Procedure for data collection

Patients were assessed within 48 hrs of admission, and within 24 hrs of planned discharge, and followed-up between two and 3 months following discharge home, at their medical check-up appointment, or by phone. After initial assessment, patients were randomly allocated to the intervention or control group using concealed allocation. A blinded research assistant assigned and recorded the patients using a computer-generated randomisation sequence, in varying block size. While patients were informed that they would be allocated to *either* the APEP or control group and upon allocation, they were neither explicitly informed nor encouraged to ask about their allocation.

Patients who had not begun the exercise sessions before withdrawal, transfer or discharge, were replaced, using the same process as above. Patients who began the exercise sessions before withdrawal from the study were not replaced. To prevent contamination, it was planned that patients who were in the same room but allocated to different exercise groups, would complete their exercise sessions in different locations separately. However, this event never occurred. Therefore, all patients were treated by their bedside. The discharge and follow-up assessments were completed by a blinded research physiotherapist.

An adverse event included a fall, cardiac ischaemia or pulmonary embolism during exercise, or an exacerbation of a condition as a result of the intervention (e.g., exacerbation of painful joints). Death or admission to intensive or critical care were considered as Serious Adverse Events. In the occurrence of adverse events, the Sponsor’s Clinical Research Supporting Officer, the Hospital Risk Manager and the treating consultant were informed. All the necessary hospital procedures and documentation were completed.

### Deviations from the published protocol

There were four significant deviations from the previously published protocol [18]. First, accelerometer-recorded walking activity was collected on a considerably lower number of patients than planned. Second, the trial was terminated early, due to a change in discharge procedures, with 190 patients of the planned 220 patients included. Third, in order to detect a deterioration in physical performance, we only recruited patients with an SPPB score of ≥2 on admission. And finally, we introduced a phone follow-up assessment for patients unable to attend a face-to-face assessment. For further details, please see Additional file 2: Appendix 2: Deviations from the published protocol.

### Statistical methods

All of the descriptive information is presented in Table 1. Throughout the results, means (±SD) are presented for normally distributed data and medians [IQR] are used for non-normally distributed data. Normality of their distribution was determined using histograms.

Intention-to-treat analysis was employed on the length of stay, death and readmission rates as full data was available irrespective of drop-outs. Time-to-event analysis using the Cox proportional hazards model was used to measure the effect of the APEP on length of stay (time to discharge) i.e. discharge being the event. The effects of the APEP on walking activity in hospital, and physical performance and QoL, both at discharge and at follow-up, was estimated using linear regression. Logistic regression was used to estimate the effects on falls, readmissions and deaths at follow-up, and post hoc were grouped to report the combined negative effects. These models estimated the effects of the intervention on the absolute scores, rather than the changes in scores.

For the adjusted models, the most important covariates were selected post hoc based on the results from the preceding observation study [1] subject matter expertise and clinical judgement, with each model included their corresponding baseline score. The effects of APEP on time to discharge and step-count were adjusted for age and frailty only. For all other adjusted models, similar covariates were used.

## Results

### Participant description

**Fig. 1:**
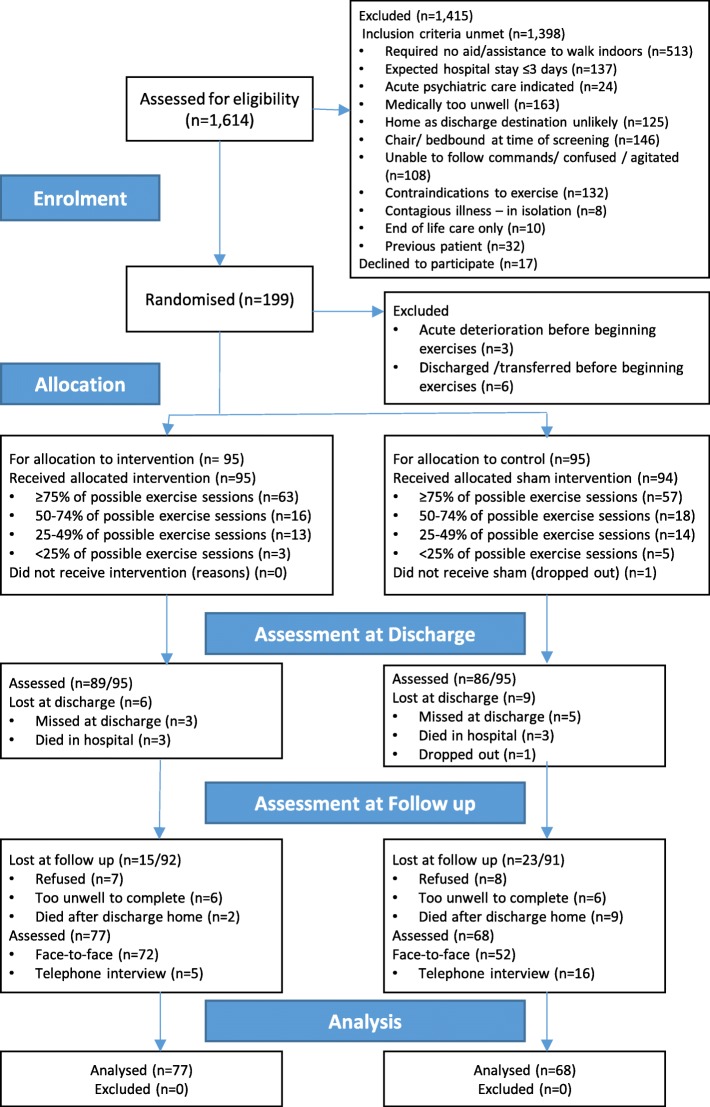
CONSORT Flow diagram of the completed APEP

During the 23-month recruitment period, approximately 5569 medical patients, aged 65 and over were admitted to the hospital. We were able to screen 1614 patients, of which 1398 did not meet the inclusion criteria and 17 declined to participate. One hundred and ninety-nine patients were randomised, and a further nine were excluded post randomisation as they failed to begin the exercise sessions. One patient dropped out from the study, leaving results from 189 patients who had completed the exercise programme for data analysis (11.7% of those screened). As per the CONSORT guidelines [32], details, including adherence, are provided in the flow diagram, (Fig. 1).

Of the 190 patients, 122 were admitted under general medical consultants, with the remainder under geriatric medicine, with similar numbers in the intervention and control groups (63 versus 59 under general medicine and 32 versus 36 under geriatric medicine respectively). Patients’ average age was 80 (±7.47) years. While the mean age differed by 2 yrs between groups, (81.6 (±7.33) in the control group and 79.9 (±7.5) in the intervention group), this was not significant (*p* = 0.07). However, all multivariable analyses adjusted for age. The only significant difference between the groups on admission was a considerably greater number of women (*n* = 61 (64%) versus *n* = 39 (41%)) in the intervention group (also included in multivariable analysis). Co-morbidity was common, with an average score of 10.15 (±3.93) on the CIRS-G and 7.4 (±3.86) medications prescribed on admission. One hundred and forty-four patients (76%) were categorised as frail, and overall, their physical performance was poor (SPPB 3.46 ± 2.06) and fear of falling high (FES-I, 46.71 ± 15.92). On admission, 39 (20%) patients walked independently with an aid, 45 (24%) needed assistance but no walking aid, and the remaining 105 (55%) patients needed both an aid and assistance. Most patients had more than one presenting complaint. In total, 60% had a cardiac issue and 34% had a respiratory condition (43/65 were infective), with significant overlap in these two groups. Seven participants had a minor stroke or transient ischaemic attack, and 13 had a urinary tract infection. In total, 22% of the included patients had a fall preceding or precipitating admission; this was the only issue in a small proportion of these (9/45). Further patient characteristics are provided in Table 2.

**Table 2:** Baseline Characteristics of the APEP Participants (*n* = 190)

Variable	Control Group (*n* = 95)	Intervention Group (*n* = 95)
	*N* (%)	*N* (%)
Female	39 (41%)	61 (64%)
Smoke		
*Never*	57 (60%)	55 (58%)
*Former*	26 (27%)	26 (27%)
*Current*	12 (13%)	14 (15%)
Marital Status		
*single*	21 (22%)	17 (18%)
*partner*	33 (35%)	30 (32%)
*widowed*	41 (43%)	48 (51%)
Alcohol		
*Never/Former*	74 (78%)	73 (77%)
*Current*	21 (22%)	22 (23%)
SHARE F-I category		
*Not Frail*	3 (3%)	7 (7%)
*Pre-frail*	18 (20%)	14 (15%)
*Frail*	74 (77%)	74 (78%)
No of falls		
*none*	50 (53%)	49 (52%)
*1–2*	31 (32%)	34 (36%)
*> 2*	14 (14%)	12 (13%)
IND		
*Independent Walking*	10 (11%)	14 (15%)
*Not Independently Walking*	84 (89%)	81 (85%)
	Mean (SD)	Mean (SD)
Age	81.7 (7.3)	79.7 (7.5)
BMI (kg/m^2^)	26.8 (6.8)	26.3 (6.5)
No. of medications	6.9 (3.87)	7.2 (3.9)
CIRS-G	10 (3.9)	10.3 (4)
VAS SR Health	52.9 (18.9)	56.5 (18.7)
FacA	3.49 (0.77)	3.59 (0.9)
	Median (IQR)	Median (IQR)
6CIT Score	6 (2–16)	8 (2–18)
FES-I	3.7 (2.8–4.6)	3.8 (2.7–4.6)
Walking Speed (m/s)	11.3 (7.4–17.2)	10 (7.6–14.9)
SPPB score	3 (2–4)	3 (2–5)
PreN-EADL	6 (0–11)	7 (0–13)
N-EADL	2 (0–4)	1 (0–4)

Abbreviations and possible score ranges: *CIRS-G*: Cumulative Illness Rating Scale-Geriatrics [higher score reflects greater impairment in several systems, range 0–56]; *6CIT*: 6-Item Cognitive Impairment Test [a higher score reflects a higher cognitive impairment, range 0–28]; *SHARE FI*: Survey of Health, Ageing and Retirement in Europe Frailty Index [a higher score reflects a higher level of frailty, range − 2.55 to 6.505]; *FES-I*: Falls Efficacy Scale-International [a higher score reflects a greater concern about falling, range 0–64]; *SPPB*: Short Physical Performance Battery [a higher score reflects a better physical performance, range 0–12]; *IND*: ability to walk independently on level surfaces. *PreN-EADL*: Premorbid Nottingham Extended Activities of Daily Living [a higher score reflects a better level of independence, range 0–22]; *N-EADL*: Nottingham Extended Activities of Daily Living on Admission [a higher score reflects a better level of independence, range 0–22]; *VAS SR Health (EQ 5D5L)*: Visual Analogue Scale EuroQol 5-Domain 5-Level, [range 1–100]; FacA: Functional Ambulatory Classification on Admission [a higher score reflects a better level of ambulation, range 0–6]

Most patients were recruited on Monday (*n* = 66, 35%), with Tuesday and Wednesday recruitment numbers similar (*n* = 49 (26%) and *n* = 46 (24%) respectively). The remainder were recruited on Thursday (*n* = 29 (15%). No significance difference in recruitment-day was detected between groups (*p* = 0.86).

### Treatment Fidelity

Adherence to the exercise programme was generally good with most participants receiving greater than 75% of the sessions. The most frequent reasons for non-completion are reported in each group. In the control group, 25 patients declined 1–3 sessions and 20 in the intervention group declined 1–2 sessions. In the control group, nine patients were too unwell for 1–2 sessions and 16 in the intervention group were too unwell for 2–3 sessions. In the control group, 21 patients were unavailable for 1–2 sessions, and in the intervention group, 14 were unavailable for 1–2 sessions. While the last session of the day was used whenever possible for those in full isolation, it remained a strong barrier, preventing up to eight exercise sessions eight patients in the control group and three in the intervention group.

### Length of stay

**Fig. 2:**
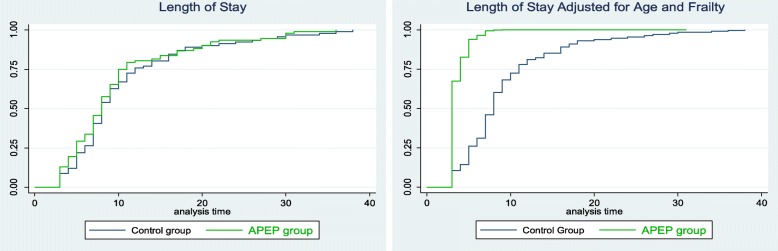
Length of Stay between Groups (*n* = 128)

The total number of bed nights for the control group was 970 (median 8 (IQR 6–13)) nights and 880 nights (median 8 (IQR 5–11)) in the intervention group (HR 1.11 (CI 0.83–1.5) *p* = 0.48). An equal number of patients were transferred to sub-acute rehabilitation in each group, which, in effect, artificially truncated their length of stay. There was little change to the results when we removed these patients (*n* = 128), (HR 1.09 (CI 0.77–1.56), *p* = 0.6; Table 3). However, when adjusted for age and frailty, while it remained insignificant, the effect was greater and, as can be seen in Fig. 2 (below), the Kaplan Meier curves become more distinctive. (*n* = 125), (HR 1.3 (CI 0.90–1.87) *p* = 0.16; Table 3).

**Table 3:** Unadjusted and Adjusted regression and Time-to-Event analyses results between groups

Variable	*N*	Control	*N*	APEP	*N*	Co-ef	Unadjusted	*p*	*N*	Adjusted	*p*
LOS *(bed nights)*	**94**	970	**95**	880	**199**	HR	1.09 (0.77–1.56)	0.6	**125**	1.3 (0.9–1.87)	0.16
Steps *median [IQR]*	**23**	597 [346–846]	**25**	889 [575–1088]	**48**	β	262.1 (−80–604)	0.1	**48**	316 (−25–656)	0.07
SPPB Score *Discharge m (±SD)*	**89**	3.7 (±2.1)	**86**	4.6 (±2.5)	**178**	β	0.88 (0.20–1.57)	0.01	**174**	0.78 (0.28–1.29)	0.003
SPPB Score *Follow-up m (±SD)*	**52**	4.7 (±2.53)	**72**	5.12 (±2.38)	**123**	β	0.45 (−0.43–1.33)	0.3	**122**	0.67 (−0.74–0.87)	0.87
VAS SR Health *Discharge m (±SD)*	**89**	62.4(±21.31)	**86**	67.7 (±18.38)	**178**	β	5.3 (−0.6–1.11)	0.07	**172**	5.10 (− 0.78–10.98)	0.9
VAS SR Health *Follow-up m (±SD)*	**68**	58.5 (±21.6)	**77**	65.2 (±21.2)	**145**	β	6.75 (0.3–13.8)	0.05	**143**	0.26 (0.09–0.47)	0.004
READMISSION *Follow-up*	**94**	16 (17%)	**95**	30 (32%)	**176**	β	2.2 (1.1–4.3)	0.03	**172**	2.9 (1.18–5.2)	0.01
FALLS *Follow-up*	**68**	18 (26%)	**77**	12 (16%)	**189**	OR	0.6 (0.26–1.36)	0.2	**184**	0.57 (0.24–1.38)	0.2
DEATH *Follow-up*	**94**	12 (19%)	**95**	5 (5%)	**189**	OR	0. 40 (0.13–1.16)	0.08	**184**	0.38 (0.11–1.14)	0.08
IND *Follow-up*	**68**	44 (65%)	**77**	58 (75%)	**167**	OR	1.68 (1. – 3.3)	0.04	**165**	1.85 (0.82–3.6)	0.05
NEGATIVE EVENTS *Follow-up*	**94**	38 (40%)	**95**	18 (19%)	**189**	OR	0.46 (0.23–0.92)	0.03	**184**	0.43 (0.2–0.92)	0.03

LOS and Steps multivariate models adjusted for age and frailty only

All other multivariable models adjusted for age, gender, frailty, fear of falling, physical performance on admission and the baseline score

Abbreviations and possible score ranges: *LOS*: length of stay (nights); *Steps*: average daily step-count; *SPPB Score*: Short Physical Performance Battery [a higher score reflects a better physical performance, range 0–12]; *IND*: ability to walk independently on level surfaces; *VAS SR Health* *(EQ 5D5L)*: Visual Analogue Scale EuroQol 5-Domain 5-Level, [range 1–100]; *READMISSION***:** medical readmissions

### Step-count in hospital

Step-count data was collected on only 48 patients and their range of activity is wide, which considerably reduced the opportunity to detect a significant difference between the groups. The APEP group were found to be more active outside of the exercise sessions (median daily steps = 889 (IQR 575–1088) compared to the control group (steps = 597 (IQR 346–846)), (p = 0.1). The difference between the groups became greater when adjusted for age and frailty (from unadjusted increase of 262 steps to adjusted increase of 316 steps (95% CI − 25 to 656), *p* = 0.07).

### Physical performance and functional independence

At discharge, physical performance scores were better in the intervention group (4.6 ± 2.5) than in the control group (3.7 ± 2.1), (difference 0.88 (95% CI 0.20–1.57), *p* = 0.01), (Table 3). In the participants who attended the face-to-face follow-up appointment, this benefit was lost (*n* = 124) (difference 0.45, (95% CI − 0.43 to 1.33) *p* = 0.3). In order to capture information on those who did not attend, we used a self-reported functional ambulation collected by a phone call (*n* = 145), and grouped the responses into independent or non-independent walkers (assistance required/not required to walk). Simple analysis suggested a greater proportion were independent in the intervention group (*n* = 58 of the 77 patients versus *n* = 44 of the 68 patients respectively, OR 1.68 (95% CI 1–3.3), *p* = 0.04; and when adjusted for age, gender, frailty, baseline physical performance and fear of falling, the scores remained similar. (Table 3).

### QoL

While all sections of the EuroQOL-5D-5 L were completed, the VAS properties support quantitative analysis, and was therefore used in the analysis. At discharge, differences in QoL were not detected. Both simple and adjusted regression suggested no difference between groups (62.4 ± 21.3 in the control group versus 67.7 ± 18.4 in the intervention group), (difference 5.3 (95% CI: − 0.6 – 1.11) *p* = 0.07); (Table 3). However, at follow-up, the intervention group reported significantly better QoL than the control group (65.2 ± 21.2 versus 58.5 ± 21.6), (difference 6.75 (95% CI 0.3–13.8), *p* = 0.05); adjustment for age, gender, frailty, fear of falling and physical performance at baseline did not materially change the estimate difference.

### Negative events

Negative events can be described as all adverse events that occurred in the hospital and negative outcomes included deaths, falls, prolonged hospitalisation and institutionalisation. Six patients died in hospital; three in each group. At follow-up, 12 patients had died in the control group, and five in the intervention group (OR 0.40 (95% CI 0.13–1.16), *p* = 0.08). A difference in prolonged hospitalisation seemed evident at follow-up; of the six patients who had not been discharged at all, five from the control group were in sub-acute care, and one from the intervention group. Also, three patients from the control group were in long-term care, with no-one from the intervention group. Once again, a difference seemed to emerge in falls. At discharge, equal numbers had fallen in hospital; three in each group. However, at follow-up, 30 patients had fallen during the study period; 18 in the control group and 12 in the intervention group (OR 0.6 (95%CI, 0.26–1.36) *p* = 0.2). Using a combined score of these negative events, post hoc logistic regression analysis showed lower odds of negative events occurring (falls, prolonged hospital stay, long-term care admission, or death) in the intervention than the control (OR 0.46 (95% CI 0.23–0.92) *p* = 0.03).

To examine readmission rates, those remaining in hospital were excluded. In the 176 patients, 46 medical readmissions occurred in the follow-up period; 16 in the control group and 30 in the intervention group (OR 2.2 (95% CI 1.1–4.3) p = 0.03); adjusted scores (OR 2.9 (95% CI 1.18–5.2) *p* = 0.01).

## Discussion

There were three main results from this study. Firstly, the APEP programme appeared to reduce length of stay by 30% in patients who were discharged home, however, the 95% confidence intervals included the null effect. Secondly, patients’ physical performance was significantly better at discharge in the intervention group, however this improvement was lost at follow-up. Finally, while significantly more readmissions occurred in the intervention group, more prolonged hospital stays, deaths and falls and admissions to long-term care occurred in the control group.

The effect of the APEP on length of stay is similar to previous findings. Neither Siebens et al. [14] nor de Morton et al. [13] detected a shorter stay with additional exercises in hospital. Jones et al. [15] detected a difference, but only when sub-acute care was included. A previously conducted individual patient meta-analysis [16] found that the additional exercises mostly benefitted non-independently mobile patients on admission, and Jones et al. [15], also found that their exercise intervention was most effective for those with poorer physical performance on admission. Conversely, a recent study has shown that patients with poor physical performance on admission (SPPB < 4) were more likely to respond adversely to an exercise intervention [33]. The sample size in this study was not large enough to explore the influences of frailty and disability, however, future studies should plan for this analysis.

Our power calculations for this study, based on a previously completed pilot study [34] suggested that 220 patients were required. The length of stay is artificial in those transferred for continuing care. When transferred patients were omitted from the analysis, the intervention effect was greater on length of stay, but the power was weakened (*n* = 125), which may explain its failure to reach statistical significance. Future studies should consider this subgroup and if possible, sufficiently power the study to detect changes.

A significant difference in physical performance was detected at discharge in the intervention group and this is similar to the findings of Jones et al. [15]. The changes that we detected suggest that they were also clinically meaningful. The change in SPPB scores of 0.78 (adjusted scores) lies well within the estimates for clinically meaningful change of between 0.3 and 0.8, and within the estimates for substantial clinical change of between 0.4 and 1.5 [35].

QoL was statistically different between groups at follow-up; a difference of 6.7 units (range 0–100 units) was detected. While there is no clearly defined minimal clinical important change in the older population, a difference of 7 units has been defined as the cut-off point for patients with chronic pulmonary disease [36], suggesting that the difference detected in this cohort is clinically relevant.

The differences in negative events became apparent at follow-up, and when analysed together (post hoc) occurred significantly more often in the control group. One adverse outcome was detected in the intervention group; a considerably greater number of readmissions occurred (30 versus 16 in the control group). The reason for this is unclear; potentially, as a greater number of frailer patients were discharged home, they may have a natural tendency for readmissions. The study sample were very heterogeneous, future studies should consider exploring possible associations with readmissions.

While the results of this trial need to be interpreted with caution, they can be used to inform future research in this area. Firstly, it is important to conclude that these types of interventions are feasible in hospital. Secondly, many frailer patients are not discharged directly home, with the health service provide developing “step-down” pathways. The APEP programme may sit well within these services as patients recover from their acute illness. Finally, many older inpatients are robust, but remain inactive in hospital. While physiotherapists lead patients’ exercise and activity, everyone is responsible for exercise promotion. Future research should measure the effectiveness of a broader, more inclusive intervention including changes to the hospital environmental and interdisciplinary management of walking activity in hospital.

### Study limitations

There are a number of limitations in this study. Two factors may have resulted in only 12% of screened older medical patients being recruited. Firstly, we aimed to recruit frailer, more disabled inpatients as we identified these who need most assistance and guidance. However, we excluded more robust patients who have been found to benefit well from exercise interventions [33]. We recruited those needing assistance of one, but resources prevented recruitment of those needing assistance of two people, resulting in a narrow patient selection. Secondly, to maintain an adequate dosage of the APEP intervention, we recruited patients early; within 2 days of admission. However, many were too unwell to recruit within that timeframe, but gradually did improve thereafter, and became clinically, very suitable for the intervention. In future studies, the dosage, specificity of the exercise programme or simply patients’ ability to participate should be debated.

While we intended to recruit 220 patients, we decided to terminate the trial after 190 patients were recruited, when it was obvious that a newly-opened transitional care unit accepted many patients in the trial, rather than directly discharged home. This resulted in an artificially shorter LOS, and did not reflect “readiness for discharge home”. This premature termination led to our study being underpowered, leaving the exact effect of the intervention unclear. It was also noted that the intervention did not affect the transfer rate; once the clinical decision to offer sub-acute rehabilitation was made, it was difficult to withdraw this offer.

The study was powered for length of stay as the primary outcome measure, and full data was available for this analysis. However, the considerable loss of patients at follow-up analysis (15 from the intervention group and 23 from the control group), limited our interpretation of the secondary results, which would have benefitted from greater numbers at recruitment.

The improvement in physical performance at discharge was lost at the three-month follow-up. The patients were not supported after discharge, nor were their activity levels monitored. Future studies should incorporate some support and or measurement of activity post discharge. Finally, more information regarding patients’ fear of falling and frailty at follow-up may provide a better indication of changes in their self-efficacy.

## Conclusions

The results suggest that the APEP intervention is feasible, improves patients’ physical performance and QoL. Length of stay was considerably reduced, however the results remain inconclusive. Insufficient study participant numbers as a result of the new subacute care unit offsite may explain the lack of significance.

## Supplementary information

**Additional file 1: Appendix 1.** Description of APEP and Sham Exercise Programmes.

**Additional file 2: Appendix 2.** Deviations from the published protocol.

## Data Availability

The datasets used and/or analysed during the current study are available from the corresponding author on reasonable request.

## Abbreviations

6-CIT: Six-Item Cognitive Impairment Test
APEP: Augmented prescribed exercise programme
CIRS-G: Cumulative Illness Rating Scale – Geriatrics
EQ-5D-5 L: EuroQol 5-Domain 5-Level Scale
FES-I: Falls Efficacy Scale International
N-EADL: Nottingham Extended Activities of Daily Living Scale
PI: Principal Investigator
RA: Research Assistant
RPT: Research Physiotherapist
SAM: Stepwatch Activity Monitor
SHARE-FI: Survey of Health, Aging and Retirement in Europe Frailty Instrument
SPPB: Short Physical Performance Battery
