# Supplementary material for: Augmented exercise in hospital improves physical performance and reduces negative post hospitalization events: a randomized controlled trial
Source: BMC Geriatr. 2020 Feb 7;20:46. doi: 10.1186/s12877-020-1436-0 (PMC7007685; doi:10.1186/s12877-020-1436-0)
Supplement: Supplementary file 1 — Additional file 1: Appendix 1. Description of APEP and Sham Exercise Programmes. [file 12877_2020_1436_MOESM1_ESM.docx]

Appendix 1: Description of APEP and Sham Exercise Programmes

#### Augmented Prescribed Exercise Programme

The intervention group were assisted or supervised in complete strengthening, balance and gait exercises. Based on the patient’s self-reported mobility issues and objective assessment, RMC, (a senior physiotherapist specialised in geriatric care) prescribed a tailored exercise programme. The exercises were prescribed and assisted by her only. The initial treatment was kept simple and straightforward to maintain patient compliance and the intensity was increased as tolerated in the subsequent sessions. Sessions lasted between 20-40 minutes and was adapted as needed daily, depending on the patients’ ability.

Exercises were designed to improve the patient’s transfer ability, balance and walking endurance. Strengthening and balance exercises were completed at the bedside. They were lower limb strengthening exercises completed in sitting, sit to stand exercises, transfer training (bed to chair, chair to chair), and balance exercises. Infection control regulations prevented the use of weights, therefore, body weight was used as a resistance.

Most patients were able to begin with the following exercises at the bedside; in sitting, ankle pump exercises, knee extensions, marching on the spot, trunk rotation exercises, reaching and stretching in sitting. Depending on the patient’s balance ability, they were asked to pick objects up off the floor in sitting, progressing the challenge as much as possible, safely. The exercises were progressed to weight bearing exercises quickly. These included sit-to-stand exercises (using the chair height and decreasing arm support to progress the exercise), transfer practice (increasing the distance of the transfer and decreasing arm support to progress the exercise), squats in standing (reducing arm support and deepening the squat to progress the exercise), hip abduction and extension exercises (reducing arm support and increasing the speed to progress the exercise), and heel raises (using decreased arm support to progress the exercise). If safe, the exercises were then progressed to balance exercises. Patients were asked to pick items up off the floor, asked to march on the spot with eyes closed, carry a small item as they transferred from bed to chair, and they were encouraged to converse during the exercises. Exercises were progressed by increasing the number of repetitions, increasing the speed and the challenge of the exercises.

The final component of the exercise session was walking. Patients walked a distance with the physiotherapist. They were encouraged to take breaks as much as needed, rather than simply return to the bedside. Light chairs were moved to facilitate rests. Distances were determined by the physiotherapist’s perception of fatigue – breathing rate, gait pattern and walking speed. Stair climbing was not part of the walking programme for safety reasons. Aids and appliances, including walking aids and portable oxygen, were provided as needed, in consultation with the ward staff and attending physiotherapist.

Patients with limited cognitive ability were also included in this trial. If the patients found it difficult or distressing to follow the instructions at the bedside, the physiotherapist increased the walking component of the programme. Often, patients could carry out the strengthening and balance exercises off the ward, in a quiet open area of the hospital rather than at the bedside. These patients were supported and guided through the exercise programme as tolerated.

Those able to walk safely and independently were strongly encouraged to walk at least three times daily independently. Family members were encouraged to “go for a walk” with the patients during visits. Advice and education about walking, general physical fitness and performance was given to the patients and their carers as required.

#### Sham Exercise Programme

The control group completed sham exercises which mainly consisted of stretching and relaxation exercises. They were completed either in the lying or sitting position only. While the patients were encouraged to talk about their condition and exercise, none were given education, encouragement or were assisted to exercise or walk more. The exercises were not progressed but rather repeated at each session. The sessions lasted between 20-30 minutes depending on the patients’ ability.

RMC kept a register of the exercises completed as well as the total number of sessions that the patients could have possibly completed, number that were actually completed, and the reason for missed sessions such as absence from ward, refusal, medical status, or care in isolation.
