# Supplementary material for: Augmented exercise in hospital improves physical performance and reduces negative post hospitalization events: a randomized controlled trial
Source: BMC Geriatr. 2020 Feb 7;20:46. doi: 10.1186/s12877-020-1436-0 (PMC7007685; doi:10.1186/s12877-020-1436-0)
Supplement: Supplementary file 2 — Additional file 2: Appendix 2. Deviations from the published protocol. [file 12877_2020_1436_MOESM2_ESM.docx]

Appendix 2: Deviations from the published protocol

#### Walking Activity Data Collection

While every attempt was made to collect walking activity data in hospital, this data proved difficult to collect. Reasons preventing its application included poor skin condition at the ankle (n=41), refusal or data collection for less than one full day (n=15) and time and manpower limitations (n=85), allowing data collection on a total of only 49 patients (26 in the exercise group and 23 in the control group). Prioritisation was given to effective recruitment, intervention and assessments.

#### Early termination of the trial

The trial began in March 2015. Originally, it was planned to recruit 220 acute medical inpatients to the study. Power calculations suggested that 200 participants were required, with an additional 20 to allow for dropouts. However, the trial was terminated in January 2017 when 190 patients were recruited to the trial.

The aim of the trial was to measure the effectiveness of augmented exercise on length of stay in acute care, and the patients’ physical ability and quality of life at discharge. In September 2016, an off-site transitional care unit opened. Many trial participants were transferred to this unit, prior to discharge home. This resulted in their length of stay in *acute* care being truncated, and their physical performance scores (assessed at discharge from acute care) poorly reflecting their readiness for discharge home. The trial could not be continued in the transitional care unit for two reasons; the patients were no longer in acute care, and logistically, it was impossible to recruit and provide twice daily exercises to patients over two sites. Therefore, following consultation with local expert statistical support, it was decided to terminate the trial in January 2017, with 190 patients recruited.

#### Exclusion of Patients with SPPB score of ≤1 on admission

Shortly after beginning the trial, the SPPB measurements from the observation study were examined in closer detail. It was noted that most patients scored less than 5/12, (mean score = 4). Many of the patients in the observation study were independently mobile, and therefore, we predicted that the mean SPPB trial score would be lower. The aim of the trial was to measure the effects on functional decline, but if the SPPB scores were too low, it would be impossible to detect functional decline. Therefore, from that point onwards, we recruited patients who scored two or more in the SPPB only. The decision to retain all the previously collected data was made to ensure that we reached the target of 220 within the trial’s timeframe.

#### Introduction of a Phone Call Follow-up Assessment

In the early stages of the trial, it was noticed that a number of patients were being lost at follow up. There were a number of reasons why they were unable to attend. Firstly, the patient group is generally frail, so many patients were directed to attend their local GP, not the hospital, for their medical follow-up. Secondly, while we provided taxi services to attend, those who lived far from the hospital declined to attend their follow-up visit. And finally, there was a large number of patients who simply refused to attend. Therefore, if it was clearly impossible for a patient to attend the hospital for the follow-up, or if the patient refused, we decided to collect as much information as possible by phone. We asked them for verbal permission to complete a phone-call interview. The phone interview prevented the measurement of their physical performance or grip strength, however, other self-reported data was collected, including a self-reported functional ambulation. If the patient was unable to complete the interview, their next of kin was approached to provide the information.
